# EMG-Free Monitorization of the Acoustic Startle Reflex with a Mobile Phone: Implications of Sound Parameters with Posture Related Responses

**DOI:** 10.3390/s20215996

**Published:** 2020-10-22

**Authors:** Christopher L. Gowen, Prashanna Khwaounjoo, Yusuf O. Cakmak

**Affiliations:** 1 Department of Anatomy, School of Biomedical Sciences, University Of Otago, Po Box 56, Dunedin 9054, New Zealand; chris.gowen@postgrad.otago.ac.nz (C.L.G.); prash.khwaounjoo@otago.ac.nz (P.K.); 2 Medtech Core, Auckland 1010, New Zealand; 3 Brain Health Research Centre, Dunedin 9054, New Zealand; 4 Centre for Health Systems and Technology, Dunedin 9054, New Zealand

**Keywords:** acoustic, startle, reaction, response, reflex, blink, mobile, sound

## Abstract

(1) Background: Acute acoustic (sound) stimulus prompts a state of defensive motivation in which unconscious muscle responses are markedly enhanced in humans. The orbicularis oculi (OO) of the eye is an easily accessed muscle common for acoustic startle reaction/response/reflex (ASR) investigations and is the muscle of interest in this study. Although the ASR can provide insights about numerous clinical conditions, existing methodologies (Electromyogram, EMG) limit the usability of the method in real clinical conditions. (2) Objective: With EMG-free muscle recording in mind, our primary aim was to identify and investigate potential correlations in the responses of individual and cooperative OO muscles to various acoustic stimuli using a mobile and wire-free system. Our secondary aim was to investigate potential altered responses to high and also relatively low intensity acoustics at different frequencies in both sitting and standing positions through the use of biaural sound induction and video diagnostic techniques and software. (3) Methods: This study used a mobile-phone acoustic startle response monitoring system application to collect blink amplitude and velocity data on healthy males, aged 18–28 community cohorts during (*n* = 30) in both sitting and standing postures. The iPhone X application delivers specific sound parameters and detects blinking responses to acoustic stimulus (in millisecond resolution) to study the responses of the blinking reflex to acoustic sounds in standing and sitting positions by using multiple acoustic test sets of different frequencies and amplitudes introduced as acute sound stimuli (<0.5 s). The single acoustic battery of 15 pure-square wave sounds consisted of frequencies and amplitudes between 500, 1000, 2000, 3000, and 4000 Hz scales using 65, 90, and 105 dB (e.g., 3000 Hz_90 dB). (4) Results: Results show that there was a synchronization of amplitude and velocity between both eyes to all acoustic startles. Significant differences (*p* = 0.01) in blinking reaction time between sitting vs. standing at the high intensity (105 dB) 500 Hz acoustic test set was discovered. Interestingly, a highly significant difference (*p* < 0.001) in response times between test sets 500 Hz_105 dB and 4000 Hz_105 dB was identified. (5) Conclusions: To our knowledge, this is the first mobile phone-based acoustic battery used to detect and report significant ASR responses to specific frequencies and amplitudes of sound stimulus with corresponding sitting and standing conditions. The results from this experiment indicate the potential significance of using the specific frequency, amplitude, and postural conditions (as never before identified) which can open new horizons for ASR to be used for diagnosis and monitoring in numerous clinical and remote or isolated conditions.

## 1. Introduction

Dysfunctional mental health affects nearly 300 million people globally with the World Health Organization defining mood and cognitive disorders as the largest contributors to human disability [1]. The burden of diseases revolving around mental health conditions is difficult to quantify given the complexity of standards of care and recording capabilities from 2nd and 3rd world nations as well as individual reporting/withholding. Information from Europe and the United States describe global costs comprising medication, physician visits, as well as hospitalization and indirect costs such as mortality, disability, and production losses accumulate to ~1.7 trillion USD [2]. Aside from these strains, additional socio-economic impact falls on the effects generated from mental health fraud and abuse. Condition masking, abuse of prescription medication as well as disability compensation have influenced patient reporting and very well may continue to without more objective and precise methods for accurate diagnosis.

The current means to examine mental health disorders are not as easily identifiable as symptoms, for example, as physical asymmetry in stroke, but usually rely on the blend of patient history, mental, and physical status examination, and laboratory and/or neuroimaging methods to detect impairments [3,4,5,6]. However, diagnosis of mood disorders where resources are constrained may solely rely on patient reporting and invite the feigning of symptoms [1]. Because of these limitations, many leading authorities on psychiatric diagnosis such as Allen Francis, have cautioned health care professionals about the diagnostic in-/deflation in both marginally symptomatic or healthy individuals while using current self-reporting practices [3,7,8].

While combining structured interviews with patient records, and laboratory and imaging review appears to produce more accurate primary and secondary diagnoses than routine clinical methods, there is still significant controversy as to what is considered the *gold standard* towards psychiatric diagnosis as well as what is the laboratory or neuroimaging test’s *expected utility*, or the difference between benefit and cost [9].

The two major diagnostic manuals for mood disorders: The Diagnostic and Statistical Manual of Mental Disorders Fifth Edition (DSM-V) and the International Classification of Diseases provide classification systems for clinical identification which encourage self-reporting and questionnaire-literary responses [3,10]. However, these systems are objectively flawed in that their recommended methods (questionnaires) cannot control reporter/assessor bias. To assist with authenticity, biologic and physiologic surrogates of neural states have involved anomalous stress hormones, heart rate variability (HRV), blood pressure, and others have been employed to describe the nervous, cognitive, and physiologic symptoms of mental health [3,8]. Bearing in mind the development of more technological methods, the acoustic startle reflex (ASR) has also proved to be a promising approach in quantifying mental health [11].

The ASR is an aversive response which is enhanced during a fear state and is diminished in a pleasant emotional context [12]. The neuronal arcade responsible for the ASR comprises unconsciously regulated brainstem and cerebral structures where diverse conditions have been found to alter both response time as well as intensity of muscle reaction to sound [13,14,15,16,17,18,19,20,21,22,23,24,25,26,27,28,29,30,31,32,33,34]. To assess anomalous blink latencies and amplitudes of the ASR, the prominent blinking muscle, the orbicularis oculi (OO), is easily and commonly accessed using electromyogram (EMG) [34,35]. Using EMG involves specialized training and equipment (wired sensors) and have not been found to be common psychiatric practice even though “each psychiatrist has their own personal style” [3]. Although correlations of OO-ASR responses have been examined between sitting and supine conditions in a post-traumatic stress disorder population [15,23,36], sitting and standing postures were not found to be examined. Standing may not always be a possibility for some participants and these conditions may also reflect differential pathways to sound and reflex pathogenesis [31].

With EMG-free muscle recording in mind, our primary aim was to identify and investigate potential correlations in the responses of individual and cooperative OO muscles to various acoustic stimuli using a mobile and wire-free system. Our secondary aim was to investigate potential altered responses to high and also relatively low intensity acoustics at different frequencies in both sitting and standing positions through the use of biaural sound induction and video diagnostic techniques and software. Sitting and standing methods provide flexibility for the use of devices for people with disabilities. These aims may then clarify the use of the ASR to researchers, medical care providers, and scientists in using sounds and postures to differentiate populations, and/or subpopulate groups into distinct neurophysiologies. We hypothesize that we may find significant details of sound amplitudes and frequencies for use in future experimentation.

### Purpose and Goals

The ASR has been investigated to a high degree using a number of tools [35]. However, an EMG free wireless ASR tool or system has yet to be developed. Additionally, using such a system to correspond responses of the left and right eye as well as a comparison between standing and sitting postures to a range of acoustic test sets have yet to be investigated. Within these contexts, the purpose of this pilot study was to develop acoustically repeatable parameters for use in ASR investigations and to subsequently develop an acoustic response spectrum. The goals of this study was to develop an inexpensive, mobile, and clinically relevant biomedical device through the use of an application (app) to deliver a specific acoustic test set and monitor the ASR responses of the eye muscles responsible for blinking.

The outcomes of the present study may provide a detailed profile of the startle reflex which has various clinical and therapeutic significances.

## 2. Methods

### 2.1. Ethics and Environment

All subjects gave their informed consent for inclusion before they participated in the study. The study was conducted in accordance with the Declaration of Helsinki, and the protocol was approved by the Human Ethics Committee of the University of Otago (Project identification code D18/407, 11.12.2018). ASR investigations were carried out in a testing environment <65 dB under interior-overhead lighting, and the environment remained at a stable temperature (21 °C).

### 2.2. Recruitment

Thirty male subjects (Otago University volunteers) between the ages of 18 and 28 (mean 24, standard deviation (SD) 3.7) were recruited and required ~15 min of ASR collection. With each participant, the subject was briefed of the proceedings, signed the consent form, and was alternated to either sitting (*n* = 15) or standing (*n* = 15) positions.

Inclusion criteria for the study required participants to be male, between the ages of 18 and 28, and in good health. The exclusion criteria were (a) medical history of neurological disease, and (b), having active stimulants and/or depressants in their system during testing time.

### 2.3. Hardware and Software

#### 2.3.1. Mobile Sensing Platform Architecture

Due to the novelty of this method and testing equipment, we had to design and cooperate individual equipment and software in order to deliver pure and repeatable sound sets (variable in frequency and amplitude) as well as collect the blinking responses of the participants. To perform these tasks, the iPhone X and iPhone X insert earphones (Apple Incorporated, Cupertino, CA, USA) were used as the hardware platforms to both deliver the acoustic battery and collect the blinking amplitude (magnitude of the blink) and response time of each blinking reflex.

The Sound Stimulus App was created (in collaboration with CodeFluegel GmbH., Graz, Austria) to integrate the acoustic test sets from Table 1 using audio files derived from (https://www.nch.com.au/tonegen/index.html) into the 2018 iPhone X operating system (iOS11.4.1) and collect the ASR blinking data. As acoustic outputs may differ between different phones, operation platforms, speakers, and sound files, we utilized iPhone X insert earphones to limit possible cross compatibility limitations.

#### 2.3.2. Application Overview

**Figure 1:**
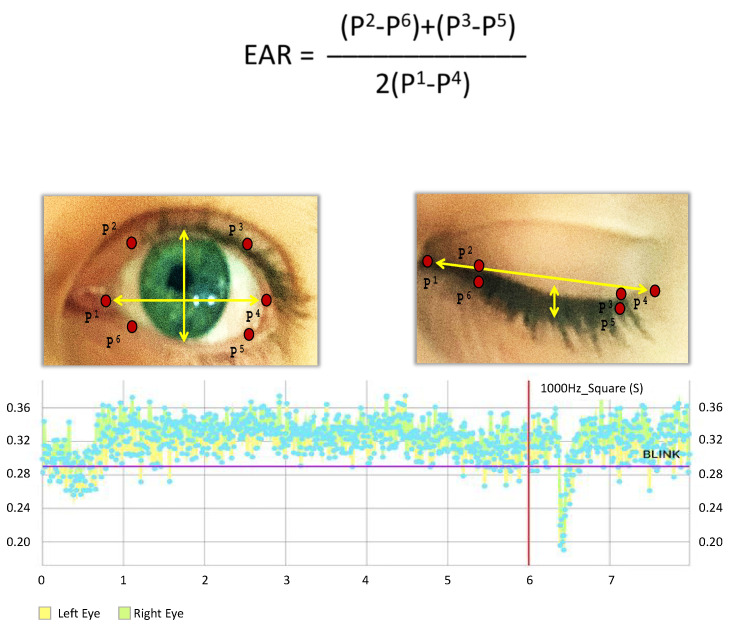
Blink–reflex detection using eye aspect ratio (EAR) across time and the geometric anchors (P1–6). The equation that provides an output for the eye size and hence acts as a blink marker based on Soukupová and Čech (2016).

In order to collect blinking data, the Sound Stimulus App identified the eyes of an individual and geometrically designated points along the eyelids to measure the movements between geometric anchors (P1–6) against others across time. This method enabled the ability to draw parameters describing the blink completeness as well as the reaction time of both eyes to each test set. The app uses a data computing Dlib library and the included default face landmarking model file [37]. Dlib is a modern C++ toolkit containing machine learning algorithms and tools for creating complex software in C++. This model provides 2D facial feature points when applied on a camera stream containing a human face. In Figure 1, we removed unwanted feature points and maintained only the eyes (6 points for each eye). The equation in Figure 1 provides an output for the eye size and hence acts as a blink marker:

The idea and formula were based on the work by Soukupová and Čech (2016), who developed a real-time algorithm to detect eye blinks in a video sequence from a standard camera [38]. We adapted this tracking method and integrated sound stimuli details within the Apple iPhone iOS.

This algorithm calculates the distances between vertical and horizontal eye feature points (one horizontal, two vertical lines) and computes the aspect ratio of acquired distances. The aspect ratio is approximately constant while the eye is opened and rapidly falls when the eye blinks. This change in EAR during a blink is used as the blink response amplitude.

#### 2.3.3. ASR Sound Stimulus Battery

The use of this novel mobile ASR monitoring system enabled the introduction of a sound stimulus battery (Table 1) of 15 pure acoustic sounds at delayed intensities using amplitudes of 65, 90, 105 dB and frequency variables of 500, 1000, 2000, 3000 and 4000 Hz. Sound sets were relative to normal human hearing ranges (1–20,000 Hz) introduced high- (4000 Hz) and low- (500 Hz) pitches/frequencies from parameters adapted from previous studies [31,33,36]. The sound sets we developed included novel use of the 3000 and 4000 Hz frequencies as well as the 65 dB amplitude to explore more diversified sound ranges beyond those historically used in literature for ASR elicitation and muscle response monitoring.

To measure the dB output of our mobile ASR monitoring system, 500, 1000, 2000, 3000, and 4000 Hz sounds were selected at 100% (Sound Stimulus App specific) volume for each test set delivery. The side buttons on the iPhone X controls the volume of the speaker (volume scales) which we had to manually select for each test set to deliver either 65, 90, or 105 dB.

We used a Digitech professional sound level meter (SLM) and a sound level calibrator (Harman International Industries, Salt Lake, UT, USA) to monitor the loudness of the testing environment as well as authenticate the dB output from the insert earphones. To accomplish this, the SLM was configured to record the highest dB output, set to “C” weighting (for checking the low-frequency content the sound), and set to “Fast” for normal measurements (fast varying noise) sound recording prior to each use. We obtained laboratory acoustic background noise using the SLM throughout a workday and measured the highest dB readings in the laboratory with the door closed to be <65 dB with the SLM placed 1 m from the door 1 m high.

### 2.4. Experimental Protocol

**Figure 2:**
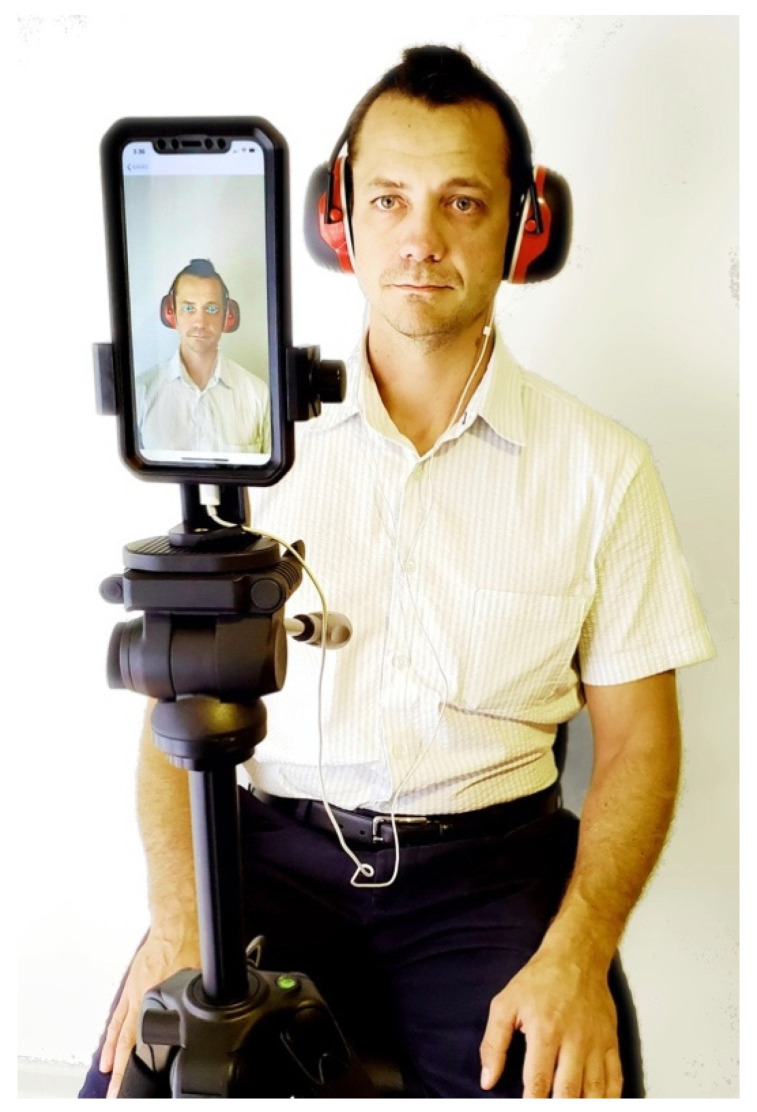
Seated volunteer with Sound Stimulus App, iPhone X, insert earphones, and noise reduction cups.

For both postures, the mobile device was placed at eye level on an adjustable tripod ~30 cm away from the volunteer’s face for optimum ASR measurements (Figure 2).

During this time, the app was set to record maximum frames per second (120 fps) for increased data collection. The participant inserted the earphones and placed over the ears further insulating (–28 dB) noise reduction cups (Work Force Maxi Muffs, Maxisafe, New South Wales, Australia) prior to the stimulus delivery.

The variables: Hz, dB, delay, and volume scale for each test were manually inputted for each delivery. Following input settings and initiation of the application, the camera begins recording. After establishing these stimulus parameters, the volunteer was instructed to remain still, and look at eye level with the iPhone X until the stimulus delivery and recordings were concluded.

**Figure 3:**
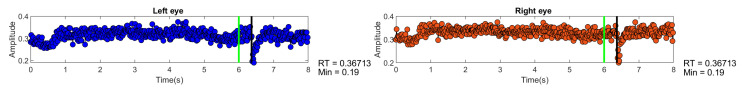
Display of eyelid geodynamics across collection time (~10 s). Left eye (blue) and right eye (red) response amplitudes and velocities. RT = Reaction time of blink reflex to acoustic stimuli, Green line = ASR sound stimulus, Black line = Blink reflex.

The sound stimulus battery encompasses a set specific order of acoustic signals which were initially randomized to determine delay and sequence (Table 1). Each of the 15 acoustic test sets (delivered as 100 ms square-wave sounds) were delivered biaurally to the ears of the participant who reported as comfortable throughout the ~15 min of stimulus delivery and blink reflex collection. The blinking amplitude and reaction time data from the total 30 tests were converted from the app display (Figure 3) to our data tables for analysis.

## 3. Statistical Analysis

To determine the significance of these data, each recording was transferred from the Sound Stimulus App to a laptop computer (2012 MacBook Pro, Apple Inc., Cupertino, CA, USA) and underwent statistical analysis and cross platform data interpretation using MATLAB (The Mathworks Inc., Natick, MA, USA), and Microsoft Office 365 computing (Microsoft Corp, Washington, DC, USA). SPSS (IBM Corp, New York, NY, USA) was utilized for data management as well. Pairwise comparisons using two tailed *t*-tests and a repeated measures one-way ANOVA for the responses for both left/right eyes and sitting/standing with Bonferroni adjusted and Tukey post-hoc test were conducted.

## 4. Results

### 4.1. Eye Synchronization

The use of our mobile acoustic-startle reflex monitoring system (MARS) allowed the collection of right and left eye responses for the acoustic battery (Table 1). The activity of the eyelid geometry from the sound-initiation onset or previous blink allowed us the ability to differentiate between open alert (yellow and green readings markers up to 6 s) and blink response or closed (Figure 1).

### 4.2. Blink Reaction Time and Response Amplitudes: Left and Right Eye, Sitting and Standing

**Figure 4:**
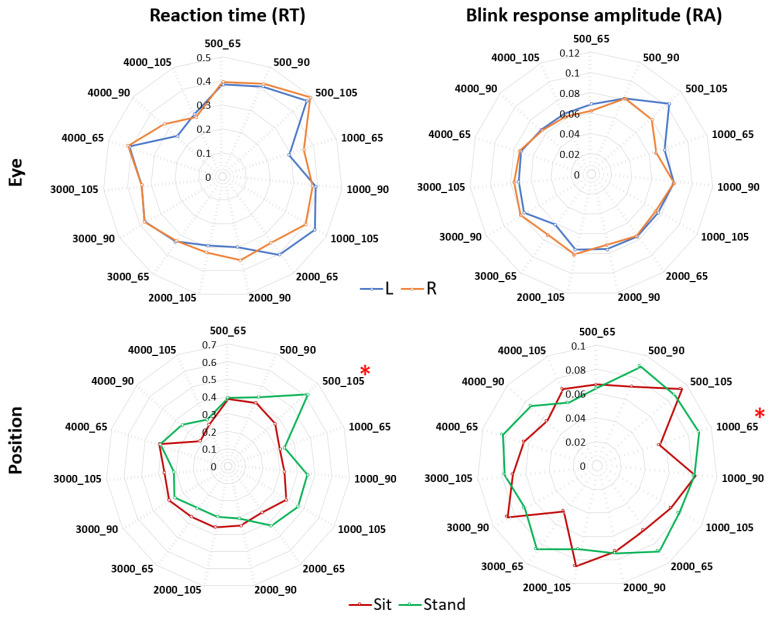
Radar plots showing the average response times (s) and amplitudes for left (L, blue) and right (R, orange) eyes and sitting (red) and standing (green) position/postures for the multiple stimuli. RT = Reaction time (s), RA = Blink response amplitude (given by change in EAR during blink). * Statistically significant *p* < 0.05.

Blink reaction times (RT) between both eyes showed no significant differences across the ASR sound stimulus battery. Whereas quickest blink reflexes for the standing and sitting postures occurred after 4000 Hz_105 dB and 4000 Hz_90 dB respectively (stand mean = 0.29 s, sit mean = 0.26 s; SD = 0.05, 0.06). The slowest RT between postures were 500 Hz_105 dB for standing (mean = 0.62 s, SD = 0.04), and 4000 Hz_65 dB for sitting (mean = 0.41 s; SD = 0.02). A comparison between sitting and standing blink reaction time identified a significant difference (*p* < 0.05) between these two postures post 500 Hz_105 dB stimuli (Figure 4). Additionally, left and right eye response amplitudes showed no significant differences. Averages of blinking amplitude for both eyes to the acoustic battery were significantly different (*p* = 0.01) between sitting and standing only with a stimulus of 1000 Hz_65 dB (Figure 4).

### 4.3. Response Times to Stimulus Parameters

**Figure 5:**
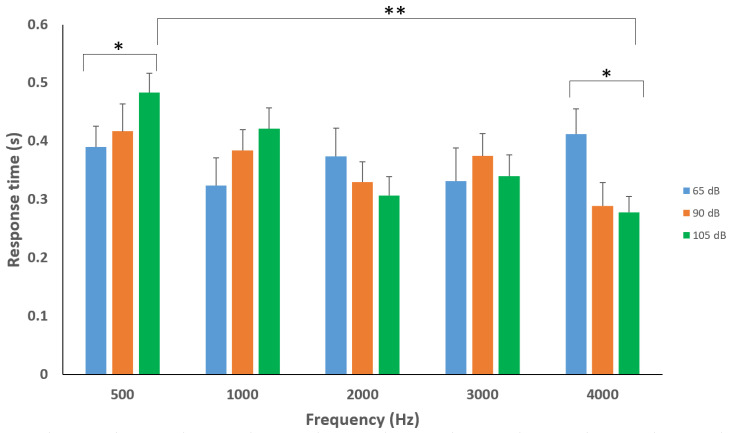
Average response times at varying frequencies and intensities. Significant amplitude-specific differences demonstrated within 500 Hz and 4000 Hz frequencies between 65 and 105 dB. Highly significant frequency-specific differences between 500 and 4000 Hz were also identified from 105 dB amplitudes. Statistically significant * *p* < 0.05, ** *p* < 0.001.

Combined pairwise comparisons at the different frequencies showed significant differences between the following test sets: 500 Hz_65 dB–500 Hz_105 dB, (*p* = 0.018); 4000_65 dB–4000 Hz_105 dB, (*p* = 0.002) and a highly significant difference (*p* = 0.000022) between test sets 500 Hz_105 dB and 4000 Hz_105 dB with the faster response at 4000 Hz_105 dB (Figure 5).

**Figure 6:**
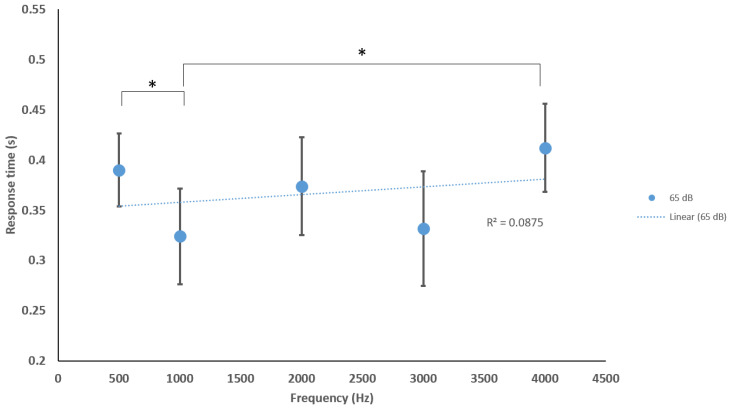
Average blink response times of sitting and standing to various frequencies at 65 dB. Significant differences were found between 500 and 1000 Hz and 1000 and 4000 Hz. R^2^ = fit of the line to the data, minimal trend in RT that can be explained by the frequency (R^2^ = 0.087). * Statistically significant *p* < 0.05.

**Figure 7:**
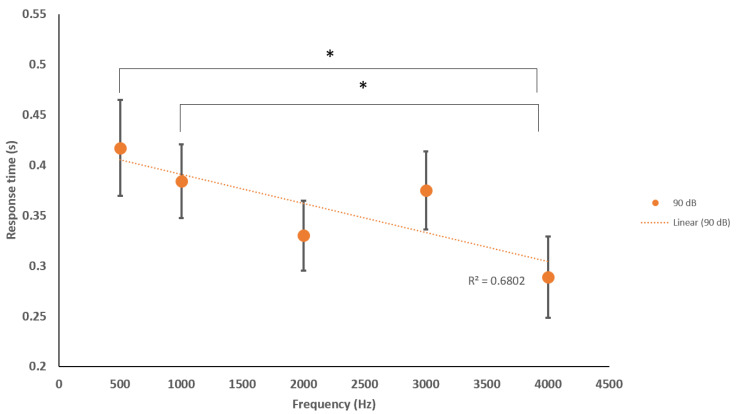
Average blink response times of sitting and standing to various frequencies at 90 dB. Significant differences were found between 500 and 4000 Hz and 1000 and 4000 Hz Indication of reduction in RT with increasing frequency, moderate linear trend (R^2^ = 0.680). * Statistically significant *p* < 0.05.

**Figure 8:**
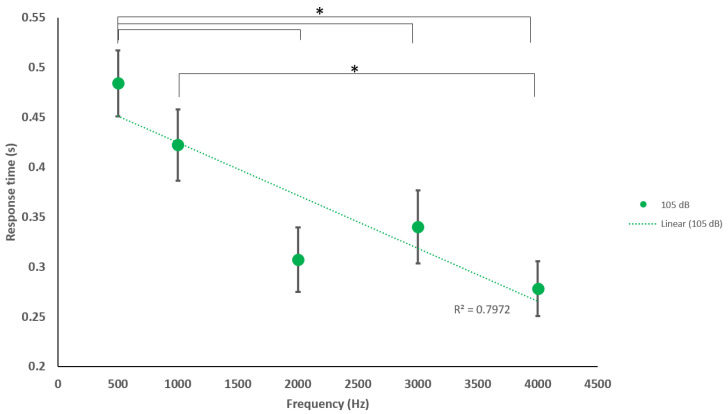
Average blink response times of sitting and standing to various frequencies at 105 dB. Significant differences were found between 500 and 2000, 3000, and 4000 Hz and between 1000 and 4000 Hz. Indication of reduction in RT with increasing frequency, moderate to strong linear trend (R^2^ = 0.797). * Statistically significant *p* < 0.05.

Additionally, we concluded the following significant differences (*p* < 0.05) between frequencies when analyzing the specific acoustic intensities of 65, 90 and 105 dB. At 65 dB: 500 Hz–1000 Hz and 1000 Hz–4000 Hz. At 90 dB: 500 Hz–4000 Hz and 1000 Hz–4000 Hz. At 105 dB: 500 Hz–2000 Hz, 500 Hz–3000 Hz, 500 Hz–4000 Hz, 1000 Hz–4000 Hz (Figure 6, Figure 7 and Figure 8).

## 5. Discussion

Using unique and historically used ASR sound sets, we were able to cue the ASR networks of healthy male participants and track the kinetics of the OO across time with a novel mobile acoustic-startle reflex monitoring system. Individual test set responses between left and right eyes were compared and showed no statistically significant differences between the reaction times or amplitudes between the eyes to any of the test sets within the acoustic battery (Figure 3). However, significant differences between the frequency response times and amplitudes after the delivery of specific sound sets were discovered. Additionally, postural conditions were found to alter the ASR network from unique acoustic stimuli.

In the standing and sitting analysis of the data, the 500 Hz_105 dB cued a faster blink reflex in the sitting posture yet the 1000 Hz_65 dB sound-set cued a stronger response for amplitude for standing (*p* < 0.05). These results were unexpected but may explain a sound set specific postural connectome in the ASR network similar to that described by [39,40], where evidence of fMRI anatomical segregation of auditory information relevant to recognition and localization is processed by distinct neuronal populations. However, these postures have yet to be evaluated using any sound stimuli while using fMRI. The combined overall quickest and slowest blink reflexes were identified at 4000 Hz_105 dB, and 500 Hz_105 dB respectively (Figure 5). These were both high- and low-pitched “loud” sounds which in non-human primates describe an evolutionary context with alertness and our relations to these sounds to the (potentially dangerous) environment [41,42]. Significant differences in blink reaction time and amplitude were determined from comparing sitting and standing postures after the delivery of the 500 Hz_105 dB and 1000 Hz_65 dB test sets respectively (Figure 4). Because these results show blink reaction time to be quicker at the 500 Hz_105 dB and a lower blink reflex amplitude at 1000 Hz_65 dB test sets in the sitting condition, these stimuli may be specified to investigations in subjects unable to stand or participants in unique environments (zero gravity or underwater environments) to address strength of response (over/underreaction) and the speed of ASR network (hyper-/hypoarousal). Furthermore, these sound sets may be expanded to additional frequency-decibel variants.

Between both postures, pairwise comparisons for the different frequencies concluded significant differences between the following test sets: 500 Hz_65 dB–500 Hz_105 dB (*p* = 0.01), 4000 Hz_65 dB–4000 Hz_105 dB (*p* = 0.02) and 500 Hz_105 dB-4000 Hz_105 dB (*p* = 0.000022). These unique sound sets may shift attitudes towards testing toward the higher and lower frequency (±4000 and 500 Hz) with different decibel ranges for future investigations, contrary to previous ASR literature using 1000 and 2000 Hz frequencies for induction. The differences in reactions to these sounds may be significant in terms of cueing the unconscious regions of reactive “survival” limbic, reticular, and autonomic systems for investigating neuronal operating bands in both healthy and disordered (hypo/hyperactive) states. Additionally, these unique sounds may establish further population-specific common ranges of activity such as those identified in children with autism spectrum disorders where hyperreactivity to weak acoustic stimuli and prolonged acoustic startle latency were found in a specific age and condition (autism) cohort [32]. Using varying frequencies and sound intensities i.e., low (500 Hz,65 dB) and high (4000 Hz, 105 dB) to test the ASR, may alter the level of pleasantness/arousal of the sound, which in the past has been associated with emotional and affectional contexts and in turn responses [43,44,45,46]. Due to these parameters causing either a very quick or slow blink reflex, investigators may utilize these tones in populations associated with neurotone hyper-/hypoarousal such as anxiety and depression [30,44], or may look at specific dysfunction (brain injury) in autonomic, reticular, limbic, or other networks intrinsic to sound processing and reflex outputs [47,48].

We found additional significant differences (*p* < 0.05) at 65 dB: 500 Hz–1000 Hz and 1000 Hz–4000 Hz. At 90 dB: 500 Hz–4000 Hz and 1000 Hz–4000 Hz. At 105 dB: 500 Hz–2000 Hz, 500 Hz–3000 Hz, 500 Hz–4000 Hz, and 1000 Hz–4000 Hz for our sound sets. These correlations are not yet understood but may represent connectome-specific tonotopy thresholds or transitions between specific frequency groups of stereocilia or ASR processes of deeper brain centers [48,49,50]. Nevertheless, using the ASR to describe more neurophysiologic spectrums of arousing and depressing activity within a reflex latency scale has applicability in defining neurological tone in patients/participants suffering from central nervous system trauma (traumatic brain injury, stroke) and neurological degenerative disorders (Alzheimer’s and Parkinson’s). For example, we know that we should see a normal range of blink, amplitude, and eye synchronicity responses from a young healthy male, but, if he were to suffer a concussion or brain injury, the ASR networks may display anomalous responses [51]. The ASR may be delayed, accelerated, or show no reflex at all during the peri/post recovery period and, during this time, the blink reflex and acoustic-processing networks may be conveniently monitored with a mobile phone before/during/after clinical examination for functional abnormalities. Further investigations incorporating the subject’s emotional state, arousal or attention, and comfort level may better correlate more specific outputs within a psychiatric context. From our analyses, there is an indication that both higher and lower frequencies with high sound amplitudes may be suitable for future studies in fields of psychiatry, specifically, anxiety, depression, and post-traumatic stress disorder (PTSD).

In this study, we created a mobile acoustic startle response monitoring app and showed that the app produced certain frequency-decibel sound sets in order to record the ASR for blink amplitude and latency in healthy adults for sitting and standing postures. We were able to identify and investigate potential correlations in the responses of individual and cooperative OO muscles to various acoustic stimuli using a mobile and wire-free system. Additionally, we found that certain sound sets induced contrasting reaction times in and between both postures and identified specific pure sounds for future startle response investigations as well as established a baseline ASR spectrum of responses for healthy adults. These healthy parameters may further be contrasted against future ASR spectrums for disordered mood and/or neurological condition monitoring.

Additionally, our findings may also lend to the classification of and recording of neurodegenerative and abnormal neurosystem conditions (such as paraplegia, cerebral palsy, or multiple sclerosis), using unique sound sets where sitting or standing postures are altered or not always possible. The ability to remotely and conveniently monitor for ASR-spectrum deviations and establish individual normative ASR reaction time and intensity ambits may also amplify performance training/conditioning schemes or establish neurosystem measurement parameters for elite programs (Astronaut/Cosmonaut/Military special operations/forces). The means of wire-free monitoring the state of the nervous system using long established and rugged hardware (smart phone with protective case) opens horizons to remote monitoring in extreme environments.

The results from this investigation indicate the potential significance of using specific frequency, amplitude, and postural conditions in ASR studies in addition to diversifying the remote monitoring capabilities of biometric devices from smart devices (iPhone). Utilizing sound parameters with posture related responses while monitoring the acoustic startle reflex with a mobile phone may open new horizons in ASR monitoring across a multitude of populations in order to identify biometric parameters of healthy responses.

### 5.1. Limitations

This was a pilot study, hence the sample size (N) was relatively small; nevertheless, our investigations were able to show usability and effectiveness in determining differences in responses to acoustic battery and posture. Future studies will utilize a larger N. Although the acoustic batteries used to induce the ASR were more diverse than those used in previous literature, time and resources limited the use of additional frequency and amplitude investigational acoustic sets, notably the use of more numerous higher or lower frequency sounds (>500 Hz and <3000 Hz). Age, sex, sleep schedule, and emotional state have been shown to affect ASR to certain degrees: lower response magnitude in aging, pre-pulse inhibition anomalies in sleep deprivation, and hyper/hypoaroused systems in emotion. However, blink response latency to diverse acoustic stimuli in these states is largely undescribed. While limitations of this study were considerations within these behavioral, mood, sleep, sex, and stress hormone variables at the time of testing, our focus and resources remained on the ASR delivery and capture system (MARS) with utility considerations of sitting and standing. As such, to reduce the variability within this pilot study, we utilized a specific age and sex cohort.

### 5.2. Comparison with Prior Work

To the best our knowledge, we are the first group to produce a mobile phone based device for ASR monitoring; hence, other direct comparisons to such systems were difficult to find in the literature. In this context, limited comparisons with EMG based ASR studies [25,26,27,28,29,30,31,32,33,34,35,36] were made. The average blink response times of the present study at 2000 Hz in standing and sitting were close to and corresponded with literature utilizing similar intensities and frequencies (90–105 dB and 2000 Hz respectively) [11,31] in the context of electromechanical delay, response latencies, response duration, late responses [11,31] and also considering the EAR threshold based ASR methodology of the present study.

These ASR latencies have the potential to be used as a diagnostic or monitoring adjunct which uses left and right eye responses to determine the presence or severity of brain injury victims [51]. Alternate frequencies outside of 2000 Hz were not identified in scientific literature and are considered novel.

## 6. Conclusions

We found significant details in the responses of cooperative OO muscles to various acoustic stimuli and identified altered responses to high and low intensity acoustics at different frequencies in both sitting and standing postures. Results suggested substantial links between individual differences in frequency, amplitude, sitting, and standing. However, further research is needed to disentangle the specific nature of these associations to one another as well as identify arousal and comfort relationships to specific frequency and amplitude acoustic test sets. To our knowledge, this is the first mobile phone-based monitoring system used to detect and report significant ASR responses to a variety of frequencies and amplitudes in sitting and standing postures. Therefore, it should be kept in mind that further replications are needed to contrast the present techniques and results or address any other potential topics. By visually recording through the use of a smart phone app, we demonstrated that it can be possible to detect and monitor the ASR in healthy population through the use of a mobile device. This opens new horizons for the ASR to be used for diagnosis and monitoring in numerous clinical conditions (e.g., stroke, traumatic brain injury, and mood disorders). The findings in the present study suggest that MARS is a simple and mobile methodology used to study the links between acute acoustic variables and their subsequent effects on the human blink response.

## Figures and Tables

**Table 1:** ASR sound stimulus battery.

Test Set	Frequency	~Decibels	Delay to Startle (s)	Volume Scales (Phone)
1	500	65	5	2
2	1000	90	6	10
3	500	105	8	12
4	500	90	5	7
5	1000	65	8	4
6	2000	65	7	3
7	4000	90	7	10
8	2000	90	7	8
9	4000	105	5	14
10	3000	105	1	12
11	4000	65	3	3
12	1000	105	7	14
13	3000	65	8	3
14	2000	105	4	12
15	3000	90	9	12

## Abbreviations

ASR	Acoustic startle reflex
OO	Orbicularis oculi
EAR	Eye Aspect Ratio
EMG	Electromyogram
SD	Standard Deviation
DSM-V	Diagnostic and Statistical Manual of Mental Disorders Fifth Edition
Hz	Hertz
dB	Decibels
N	Sample size
SLM	Sound level meter
MARS	Mobile acoustic-startle reflex monitoring system
RT	Reaction time
P	Probability value
